# Supplementary material for: Transcriptionally active enhancers in human cancer cells
Source: Mol Syst Biol. 2021 Jan 27;17(1):e9873. doi: 10.15252/msb.20209873 (PMC7838827; doi:10.15252/msb.20209873)
Supplement: Supplementary file 1 — Appendix [file MSB-17-e9873-s001.pdf]

Appendix to *Transcriptionally active enhancers in human cancer cells*

Katja Lidschreiber, Lisa A Jung, Henrik von der Emde, Kashyap Dave, Jussi Taipale, Patrick Cramer, and Michael Lidschreiber

Contents

1 APPENDIX FIGURES S1-5 **2**

Appendix Figure S1 **2**

Appendix Figure S2 **5**

Appendix Figure S3 **6**

Appendix Figure S4 **7**

Appendix Figure S5 **9**

2 APPENDIX TABLE S1: Number of cells per experiment **10**

3 APPENDIX TABLE S2: Sequences of guide RNAs and primer pairs **10**

4 REFERENCES **11**

1 Appendix Figures S1-5

Appendix Figure S1

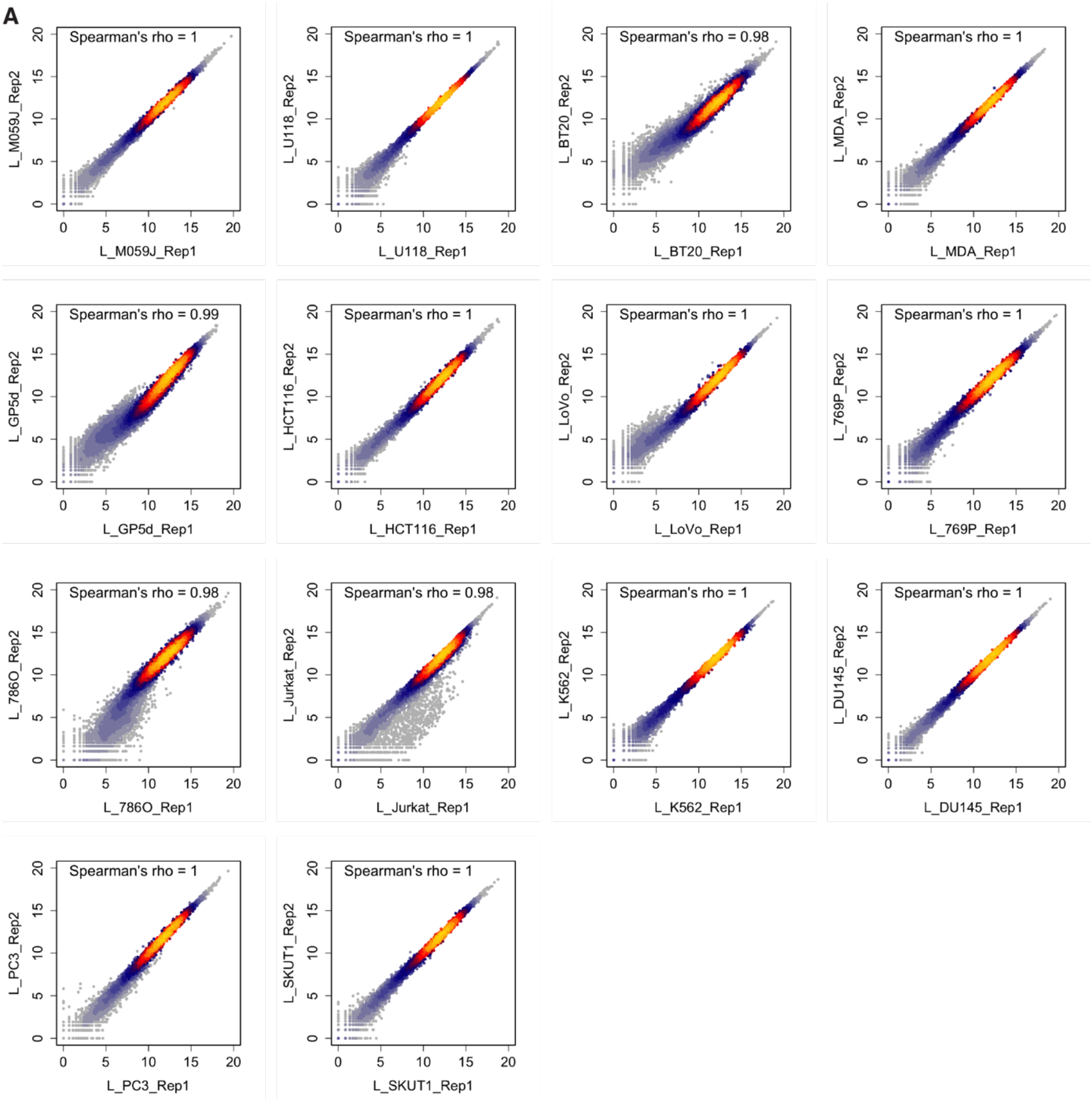

B

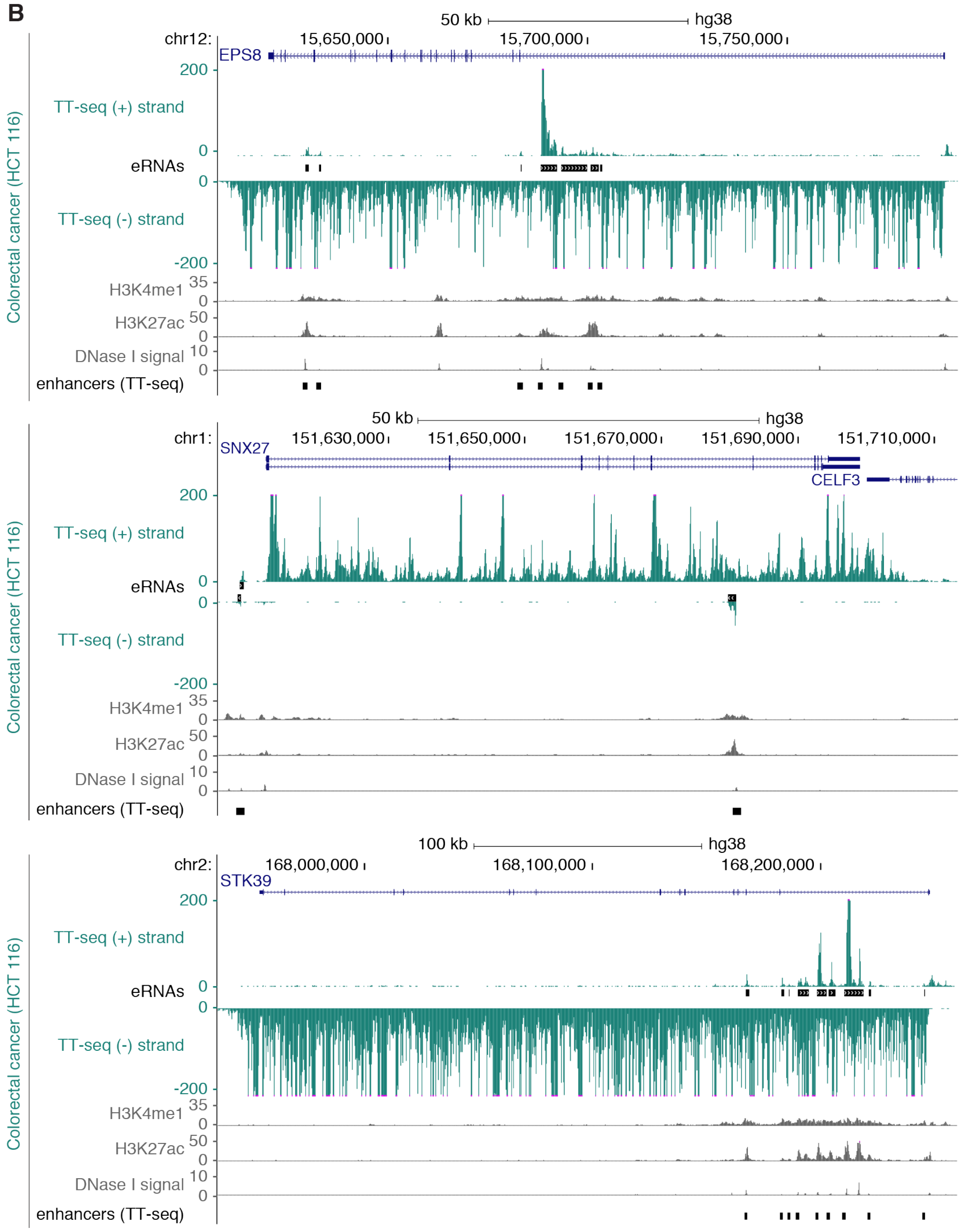

50 kb
hg38
chr12:
15,650,000
15,700,000
15,750,000
EPS8
Colorectal cancer (HCT 116)
200
TT-seq (+) strand
0
eRNAs
0
TT-seq (-) strand
-200
H3K4me1
35
0
H3K27ac
50
0
DNase I signal
10
0
enhancers (TT-seq)
50 kb
hg38
chr1:
151,630,000
151,650,000
151,670,000
151,690,000
151,710,000
SNX27
CELF3
Colorectal cancer (HCT 116)
200
TT-seq (+) strand
eRNAs
0
0
TT-seq (-) strand
-200
H3K4me1
35
0
H3K27ac
50
0
DNase I signal
10
0
enhancers (TT-seq)
100 kb
hg38
chr2:
168,000,000
168,100,000
168,200,000
STK39
Colorectal cancer (HCT 116)
200
TT-seq (+) strand
0
eRNAs
0
TT-seq (-) strand
-200
H3K4me1
35
0
H3K27ac
50
0
DNase I signal
10
0
enhancers (TT-seq)

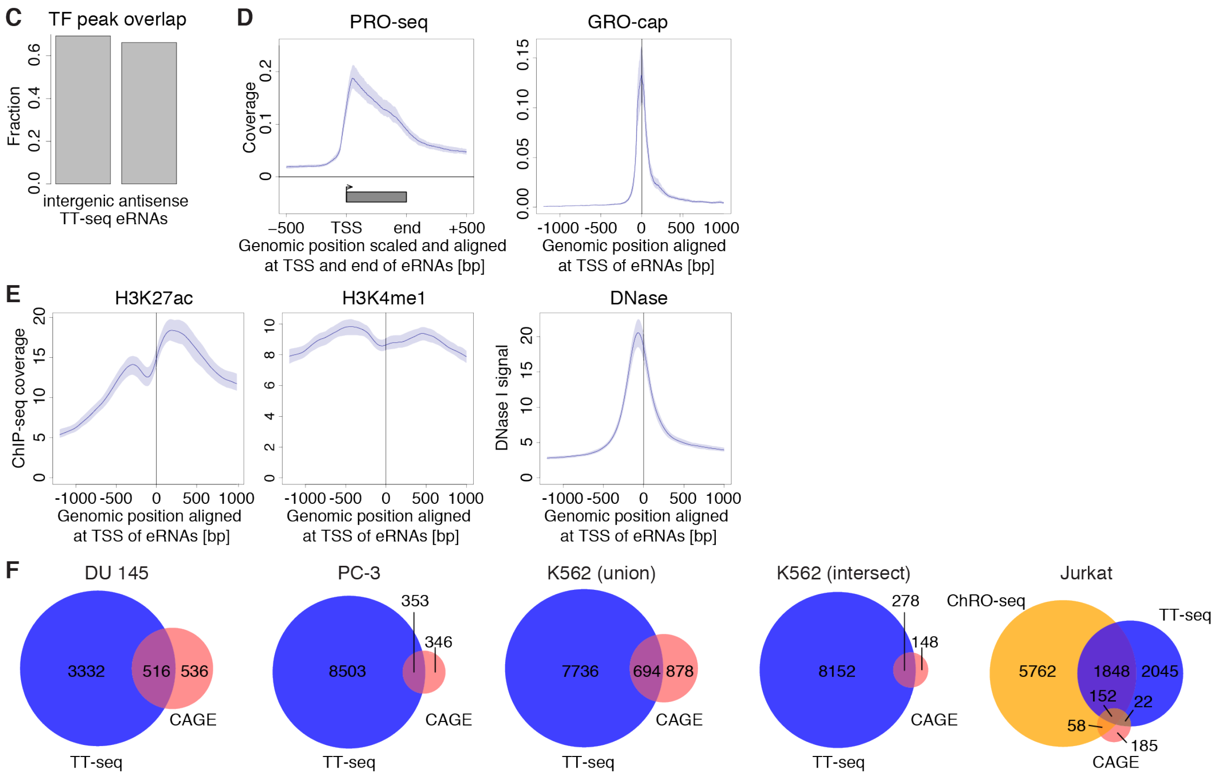

Appendix Figure S1: Mapping enhancer transcription in human cancer cells.

(**A**) TT-seq data sets are highly reproducible. Scatter plots comparing TT-seq log2 fragment counts for protein-coding genes (RefSeq) between biological replicates. Spearman correlation > 0.98.

(**B**) Antisense eRNAs mark intragenic candidate enhancer regions. Top: UCSC genome browser view of normalized TT-seq coverage on the plus and minus strand at the *EPS8* locus (hg38; chr12:15,607,591-15,792,681) in HCT 116 colorectal cancer cells. TT-seq coverage is cut at 200 (purple lines) to allow for better visualization. eRNAs are highlighted between the plus and minus strand, TT-seq enhancer regions are highlighted at the bottom. H3K4me1 ChIP-seq, H3K27ac ChIP-seq and DNase I-seq signal from ENCODE (Zhang et al., 2020). Middle: *SNX27* locus (hg38; chr1:151,605,144-151,713,424). Bottom: *STK39* locus (hg38; chr2:167,935,668-168,259,860).

(**C**) Transcription factor (TF) ChIP-seq peak overlap of intergenic and antisense eRNAs. Barplots show the fraction of transcripts overlapping at least one TF ChIP-seq peak within ± 500 bp of the TSS. Exemplary data from LoVo colorectal cancer cells, for which ChIP-seq data was available for 326 TFs ((Yan et al., 2013), Methods).

(**D**) Left: Scaled metagene profile of PRO-seq signal (Core et al., 2014) showing mean signal of antisense eRNAs in K562 cells (n=5,202). The shaded area represents the 95% confidence interval of the mean. The metagene window was expanded 500 bp upstream and downstream. The PRO-seq signal between TSS and end of the annotated transcribed unit was scaled by binning. Right: Metagene profile of GRO-cap signal (Core et al., 2014) aligned at the TSS of antisense eRNAs in K562 cells (n=5,202). The blue line shows the mean signal and the shaded area represents the 95% confidence interval of the mean.

(**E**) Metagene profiles aligned at the TSS of antisense eRNAs in K562 cells (n=5,202). Data taken from ENCODE (Dunham et al., 2012). The blue line shows the mean signal and the shaded area represents the 95% confidence interval of the mean.

(**F**) Venn diagrams showing the overlap between putative enhancer regions based on TT-seq and CAGE (DU 145, PC-3, K562) or based on TT-seq, CAGE, and ChRO-seq (Jurkat). For K562 CAGE data was available for three biological replicates and overlaps were calculated using the union or the

intersection of the enhancer regions called for the individual replicates. Note: Even though we did some filtering to make the enhancer annotations between the different methods more comparable (Methods), inherent differences attributed to the diverse enhancer calling approaches remain, making a detailed comparison difficult. For example, CAGE enhancers were called by the FANTOM consortium requiring relatively balanced transcription from both strands of the enhancer region (Andersson et al., 2014), whereas the dREG pipeline used to call enhancers from ChRO-seq data also identifies some unidirectionally transcribed regulatory regions (Chu et al., 2018).

Appendix Figure S2

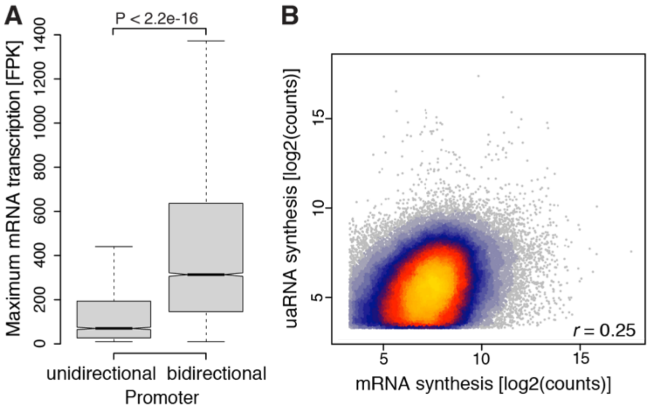

Appendix Figure S2.: Transcription directionality is preserved across cell types.

(**A**) Bidirectional promoters show higher mRNA synthesis. Boxplots showing maximum mRNA synthesis over all cell lines for unidirectional (n=2,188) and bidirectional (n=9,771) promoters. Bidirectional and unidirectional promoters were defined as having uaRNA transcription detected in at least one and none of the cell lines, respectively (Methods). Box limits are the first and third quartiles, the band inside the box is the median. The ends of the whiskers extend the box by 1.5 times the interquartile range. Notches represent 95% confidence intervals for the median values. Outliers not shown. P-value by two-sided Mann-Whitney U-test.

(**B**) Heat scatterplot comparing log2 TT-seq counts in 1 kbp regions for mRNAs and uaRNAs for all promoters in all cell lines that have at least 10 read counts in the sense and upstream antisense direction (Methods). Pearson correlation r = 0.25.

Appendix Figure S3

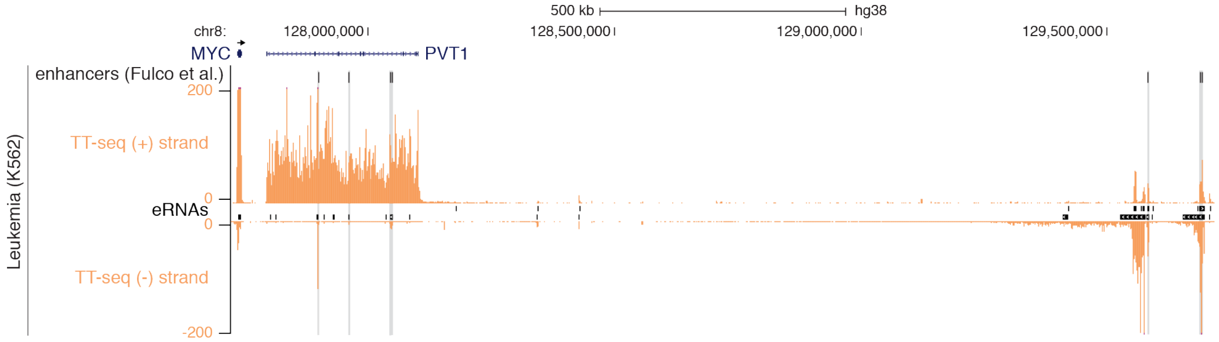

Appendix Figure S3: Transcription at functionally verified enhancers at the MYC locus. UCSC genome browser view of normalized TT-seq coverage on the plus and minus strand at the *MYC* locus (hg38; chr8:127,721,595-129,717,925; (Kent et al., 2002)) in the chronic myelogenous leukemia cell line K562. TT-seq coverage is cut at 200 (purple lines) for better visualization. eRNAs are highlighted between the plus and minus strand. Seven functionally verified enhancers activating *MYC* transcription (Fulco et al., 2016) are illustrated on top (grey lines).

Appendix Figure S4

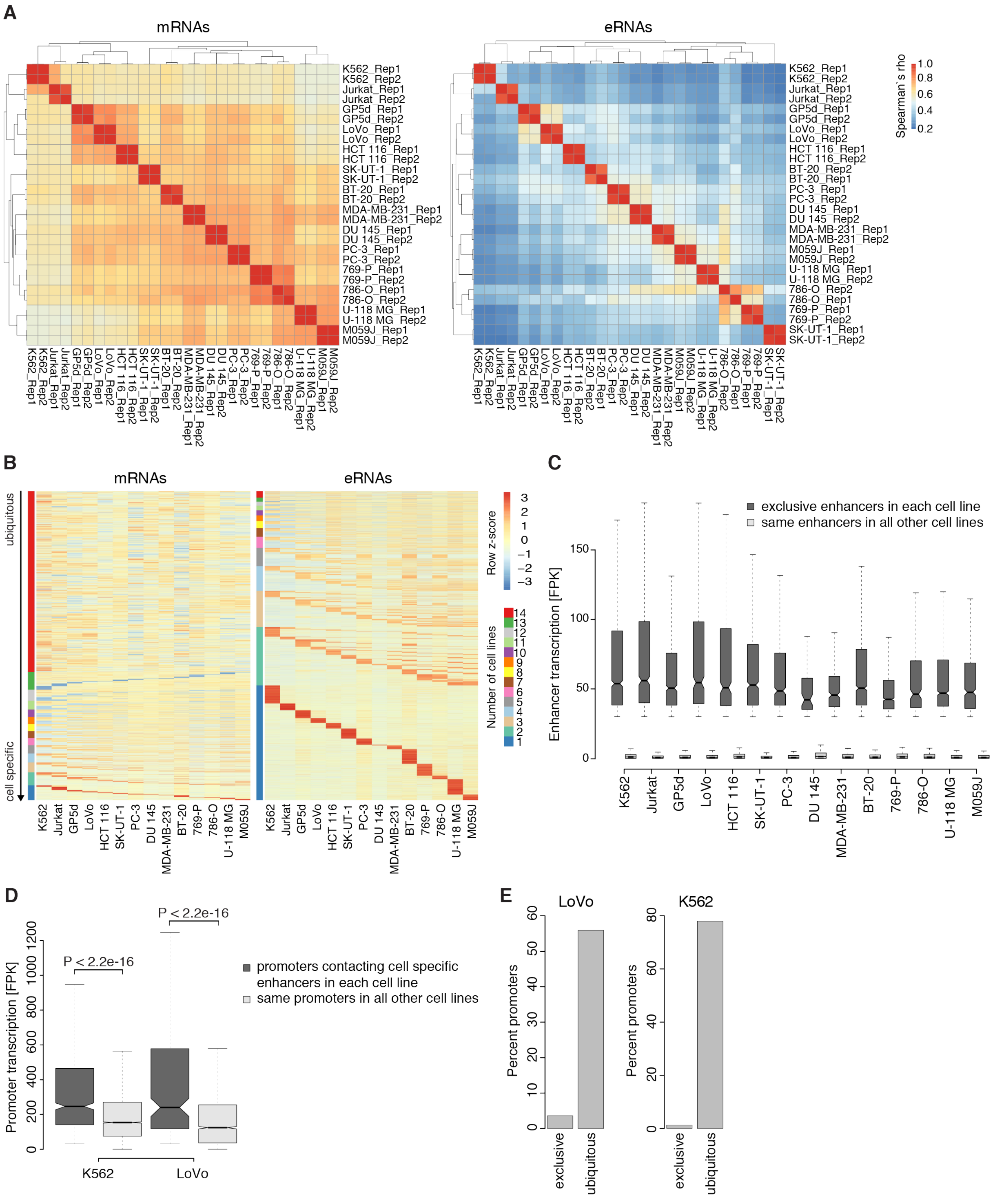

Appendix Figure S4: Cell type specificity of enhancer transcription.

(**A**) Unsupervised clustering of all pairwise Spearman correlations of normalized TT-seq signal for mRNA (left) and eRNA (right) for the union of transcripts over all fourteen cancer cell lines (Methods). Here, biological replicates are kept separately, whereas in Figure **5A** normalized TT-seq signal was averaged over replicates.

(**B**) Heatmaps showing normalized TT-seq signal for mRNAs (left) and eRNAs (right) for all fourteen cancer cell lines (columns). Heatmaps show only transcripts (rows, Z-score transformed

log2(normalized FPK + 1)) with normalized FPK >= 30 in at least one of the cell lines (mRNAs, n=14,040; eRNAs n=48,105). Heatmaps are ordered by increasing cell type specificity, as indicated by the colored bars to the left showing the number of cell lines a transcript is observed in.

(**C**) Boxplots showing normalized TT-seq signal for enhancers in the cell line in which the enhancers are exclusively transcribed compared to all other cell lines. All pairwise comparisons are significantly different (P-values < 2.2e-16, two-sided Mann-Whitney U-test). Box limits are the first and third quartiles, the band inside the box is the median. The ends of the whiskers extend the box by 1.5 times the interquartile range. Notches represent 95% confidence intervals for the median values. Outliers not shown.

(**D**) Boxplots showing normalized TT-seq signal for promoters with observed physical proximity to enhancers which are exclusively transcribed in the indicated cell line (dark grey) compared to the same promoters in all other cell lines in which the respective enhancers are not transcribed (light grey). Information regarding physical proximity was based on Pol II ChIA-PET data and promoter capture Hi-C data for K562 and LoVo, respectively (Methods). All pairwise comparisons are significantly different (P-values < 2.2e-16, two-sided Mann-Whitney U-test). Median, hinges, whiskers and notches are shown as in **C**. Outliers not shown.

(**E**) Barplots showing percentage of interacting promoters for which transcription is observed exclusively in the same cell line as the respective exclusively in this cell line transcribed enhancers (exclusive) or is observed in all fourteen cell lines (ubiquitous).

Appendix Figure S5

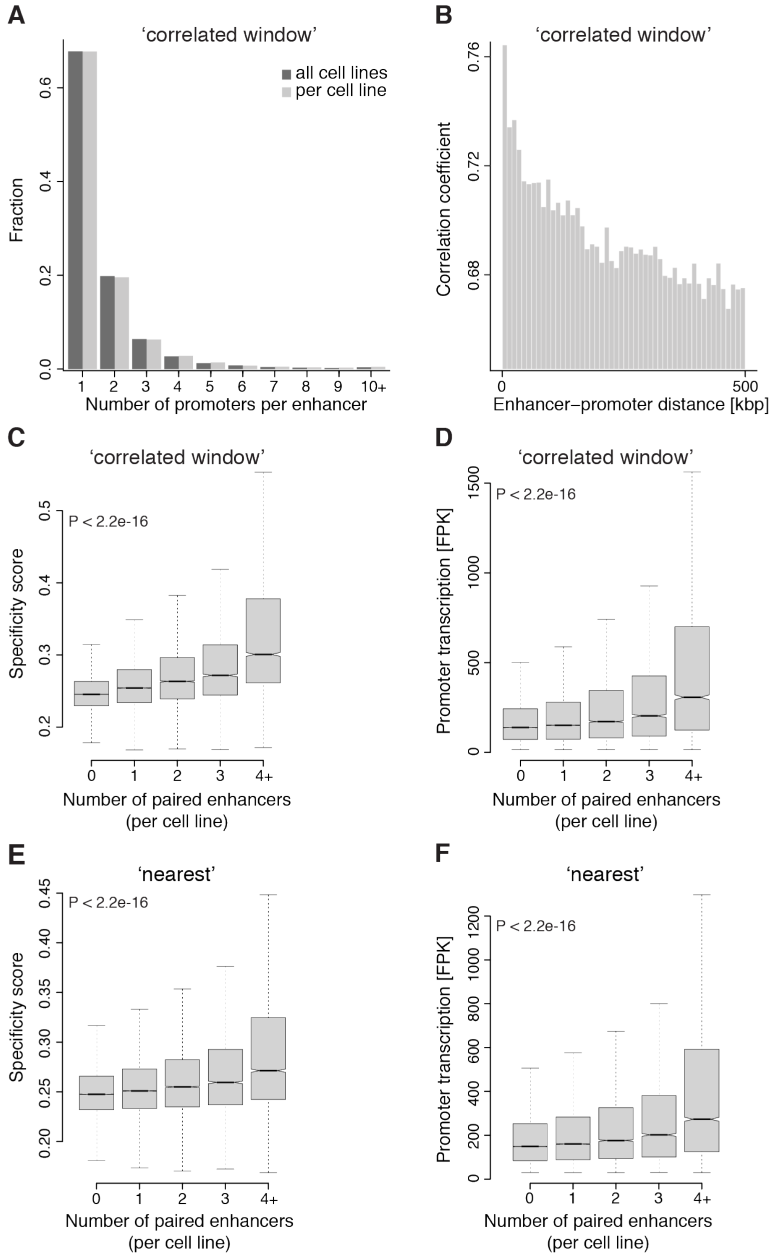

Appendix Figure S5: Enhancer-promoter pairing based on transcription correlation.

(**A**) Number of promoters per enhancer, for all enhancers that were paired to at least one promoter with the 'correlated window' approach. Fractions are shown for promoters per enhancer per cell line (light grey) and over all cell lines (dark grey).

(**B**) Distribution of average correlation coefficients between enhancer and promoter transcription depending on E-P distance. The histogram shows the 500 kbp distance range in 10 kbp bins. Only E-P pairs with correlation coefficient $r > 0.6$ ('correlated window' approach) are considered.

(**C**) As **Figure 6F** but for the 'correlated window' approach.

(**D**) As **Figure 6G** but for the 'correlated window' approach.

(**E**) As **Figure 6F** but for the 'nearest' approach.

(**F**) As **Figure 6G** but for the 'nearest' approach.

2 Appendix Table S1: Number of cells per experiment

Cancer cell line	Number of cells seeded per dish [10^6]	Number of dishes	Total number of cells [10^6] for 600 μg RNA
M0569J	1,2	10	22
U-118 MG	2,3	5	18
BT-20	4,0	6	35
MDA-MB-231	4,5	5	33
GP5d	4,5	4	36
HCT 116	6,0	2	38
LoVo	10,0	2	40
769-P	1,5	6	28
786-O	1,5	6	18
DU 145	2,5	5	30
PC-3	3,0	5	30
SK-UT-1	2,0	5	29

The number of required cells depends on cell specific parameters and is different for each cell line. Table lists the number of 15 cm cell culture dishes and cells seeded per dish 48 hours before the experiment to obtain 70 % confluent cells at the time of 4sU labeling as well as the total number of cells required to yield 600 μg total RNA for purification of 4sU-labeled RNA.

3 Appendix Table S2: Sequences of guide RNAs and primer pairs

sgRNA target	sgRNA	sgRNA sequence
Myc enhancer	Enh4-1	GTAGAATGTCAACTTCATGA
Myc enhancer	Enh4-2	ATTATATGAACTGAGAATGA
Myc enhancer	CTCF-1	TACTTTCGCAAACCTGAACG
Myc enhancer	CTCF-2	GCAAAATCCAGCATAGCGAT
Myc enhancer	Enh5dn-S2	TAGATGGGATGGCGGCCGGG
Myc enhancer	Myc335dn-S4	CTCTTCCCAGAGTAGAACAG
Myc enhancer	Myc335dn-S6	GCACCGTCTCTCCATTCCCA
Myc enhancer	Enh3dn-S8	GCACCGGTTGTGCAGCTCCA
IGH locus	IGH1	TTTCTTCGAGACCCATACCA
IGH locus	IGH2	AGACTCCTCCCTTCAGGGCA
IGH locus	IGH3	AGAGAGAAGGAGATAAGGAG
IGH locus	IGH4	ATACAAATATACATTTATAG
IGH locus	IGH5	GTCTGTGGTACAGTAATACA
IGH locus	IGH7	TCTCTCACAGACAGACACAT
IGH locus	IGH9	CCCAACACTTTGGGAAACCG
IGH locus	IGH11	GCTCTCTGTCTTCCATGCCA

Genotyping primer pairs		
Myc enhancer	Enh4PF1	TAGGGCAGTGGTTGAGAGGGTTAAG
Myc enhancer	CTCFPR1	GGAGAGGAGTATTACTTCCGTGCCT
IGH locus	IGHGeno6Fw	GGTGAAGGTCTGGCAAGTTAATCAT
IGH locus	IGHGeno6Rev	ATACATCAAAACCGAAACTGCTTTT

4 References

Andersson, R., Gebhard, C., Miguel-Escalada, I., Hoof, I., Bornholdt, J., Boyd, M., . . . Sandelin, A. (2014). An atlas of active enhancers across human cell types and tissues. *Nature, 507*(7493), 455-461. https://doi.org/10.1038/nature12787

Chu, T., Rice, E. J., Booth, G. T., Salamanca, H. H., Wang, Z., Core, L. J., . . . Danko, C. G. (2018). Chromatin run-on and sequencing maps the transcriptional regulatory landscape of glioblastoma multiforme. *Nature Genetics, 50*(11), 1553-1564. https://doi.org/10.1038/s41588-018-0244-3

Core, L. J., Martins, A. L., Danko, C. G., Waters, C. T., Siepel, A., & Lis, J. T. (2014). Analysis of nascent RNA identifies a unified architecture of initiation regions at mammalian promoters and enhancers. *Nature Genetics, 46*(12), 1311-1320. https://doi.org/10.1038/ng.3142

Dunham, I., Kundaje, A., Aldred, S. F., Collins, P. J., Davis, C. A., Doyle, F., . . . Lochovsky, L. (2012). An integrated encyclopedia of DNA elements in the human genome. *Nature, 489*(7414), 57-74. https://doi.org/10.1038/nature11247

Fulco, C. P., Munschauer, M., Anyoha, R., Munson, G., Grossman, S. R., Perez, E. M., . . . Engreitz, J. M. (2016). Systematic mapping of functional enhancer–promoter connections with CRISPR interference. *Science, 354*(6313), 769-773. https://doi.org/10.1126/science.aag2445

Kent, W. J., Sugnet, C. W., Furey, T. S., Roskin, K. M., Pringle, T. H., Zahler, A. M., & Haussler, D. (2002, Jun). The human genome browser at UCSC. *Genome Res, 12*(6), 996-1006. https://doi.org/10.1101/gr.229102

Yan, J., Enge, M., Whitington, T., Dave, K., Liu, J., Sur, I., . . . Taipale, J. (2013). Transcription factor binding in human cells occurs in dense clusters formed around cohesin anchor sites. *Cell, 154*(4), 801-813. https://doi.org/10.1016/j.cell.2013.07.034

Zhang, J., Lee, D., Dhiman, V., Jiang, P., Xu, J., McGillivray, P., . . . Gerstein, M. (2020, Jul 29). An integrative ENCODE resource for cancer genomics. *Nat Commun, 11*(1), 3696. https://doi.org/10.1038/s41467-020-14743-w
