# Supplementary material for: Transcriptionally active enhancers in human cancer cells
Source: Mol Syst Biol. 2021 Jan 27;17(1):e9873. doi: 10.15252/msb.20209873 (PMC7838827; doi:10.15252/msb.20209873)
Supplement: Supplementary file 2 — Expanded View Figures PDF [file MSB-17-e9873-s002.pdf]

Expanded View Figures

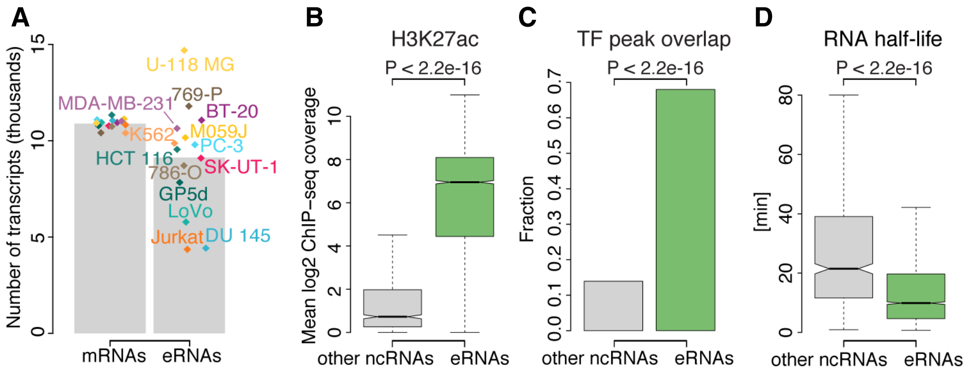

Figure EV1. TT-seq-defined eRNAs show properties characteristic of actively transcribed enhancers.

A Number of mRNAs (left) and putative eRNAs (right) per cell line. Bars depict the mean. Diamonds represent individual numbers in each cell line and are color-coded according to cancer type (as in Fig 1A).

B H3K27ac ChIP-seq coverage (Hung *et al*, 2019) at eRNAs ($n = 5{,}779$) and other ncRNAs ($n = 869$), i.e., intergenic and asRNAs, not originating from an enhancer state. Boxplots show the mean $\log_2$ coverage within ± 500 bp of the TSS in LoVo colorectal cancer cells. *P*-value by two-sided Mann–Whitney *U*-test. Box limits are the first and third quartiles, and the band inside the box is the median. The ends of the whiskers extend the box by 1.5 times the interquartile range. Notches represent 95% confidence intervals for the median values. Outliers not shown.

C Transcription factor (TF) ChIP-seq peak overlap of eRNAs and other ncRNAs (as in (B)). Barplots show the fraction of transcripts overlapping at least one TF ChIP-seq peak within ± 500 bp of the TSS. Exemplary data from LoVo colorectal cancer cells, for which ChIP-seq data were available for 326 TFs ((Yan *et al*, 2013) Materials and Methods). *P*-value by Fisher's exact test.

D Estimated RNA half-life for eRNAs ($n = 3{,}181$) and other ncRNAs ($n = 678$) (as in (B)). RNA half-life estimation was done as described (Schwalb *et al*, 2016). Exemplary data from LoVo colorectal cancer cells. *P*-value by two-sided Mann–Whitney *U*-test. Median, hinges, whiskers, and notches are shown as in (B).

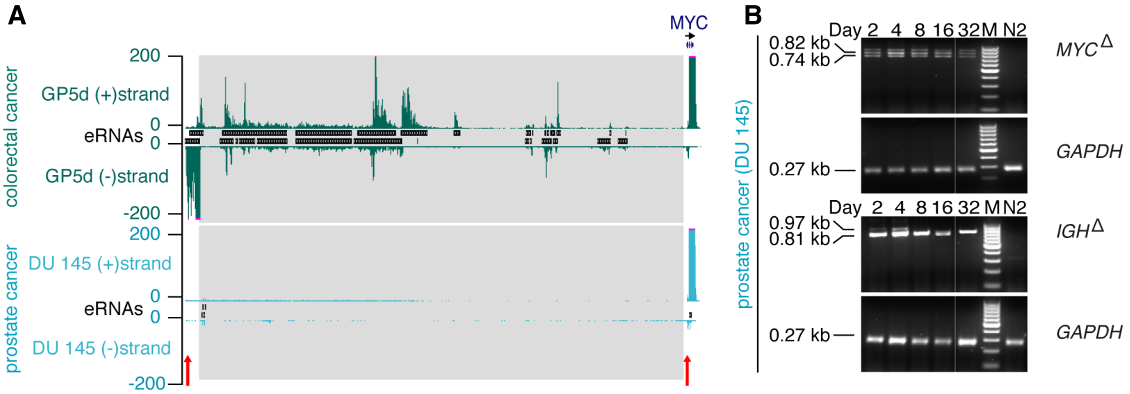

Figure EV2. TT-seq measures transcription at functionally verified enhancers.

A Normalized TT-seq coverage upstream of *MYC* in wild-type GP5d colorectal cancer cells (top) and wild-type DU 145 prostate cancer cells (bottom). CRISPR/Cas9 deletion region targeted in Dave *et al* (2017) and (B) is highlighted in gray. Red arrows mark (not to scale) the genotyping primers used in (B).

B PCR-genotyping results of the *MYC* locus and the control IGH locus in CRISPR/Cas9 edited DU 145 cells over time. GAPDH was used as internal control. M, 100-bp ladder DNA molecular weight marker. N2, non-transfected cells at day 2. See Source Data for Fig EV2 for original gel image.

Source data are available online for this figure.

 © 2021 The Authors

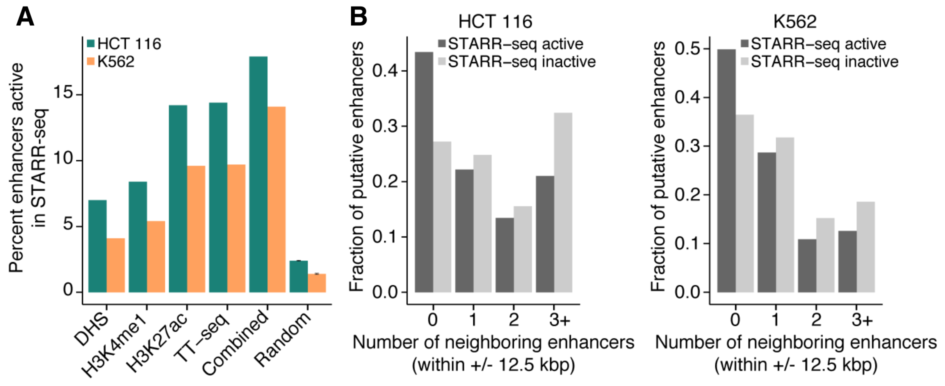

Figure EV3. Activity of putative enhancers in STARR-seq.

A Barplots showing the percentage of putative enhancers overlapping STARR-seq peaks in HCT 116 cells (green) and K562 cells (orange). Percentages are shown for enhancer regions called by DHS (n = 96,066/139,432), H3K4me1 (n = 89,670/95,272), H3K27ac (n = 28,805/41,399), or TT-seq (n = 8,407/8,430). Combined refers to enhancer regions called by DHS, H3K27ac and TT-seq (n = 5,533/4,136). Random refers to the mean observed overlap obtained using 20 sets of randomly sampled TT-seq enhancer-matched genomic regions (Materials and Methods), and error bars represent 95% confidence intervals.

B Distribution of the number of neighboring enhancers (within ± 12.5 kbp) for each candidate enhancer of the combined set (see (A)) in HCT 116 cells (left) and K562 cells (right). Shown as fractions of enhancers called active (dark gray) or inactive (light gray) by STARR-seq. Candidate enhancers inactive in STARR-seq have significantly more neighboring enhancers (two-sided Mann–Whitney U-test, P-value < 2.2e-16 (HCT 116) and P-value = 9.256e-14 (K562)).

© 2021 The Authors

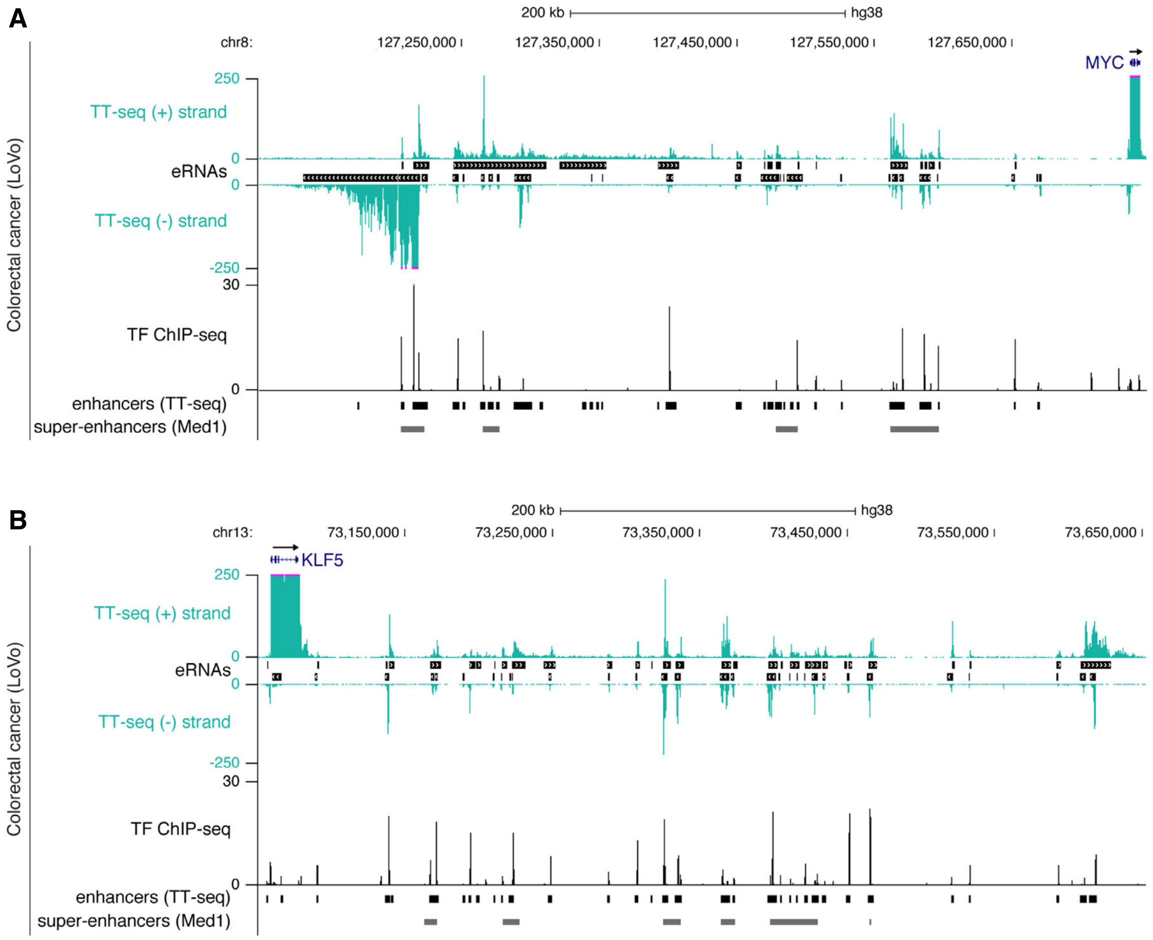

Figure EV4. Extensive eRNA synthesis in large regulatory regions.

A UCSC genome browser view of normalized TT-seq coverage on the plus and minus strand at the *MYC* locus (hg38; chr8:127,102,874–127,747,782 (Kent *et al*, 2002)) in LoVo colorectal cancer cells. TT-seq coverage is cut at 250 (purple lines) to allow for better visualization. eRNAs are shown between the plus and minus strand. Aggregated transcription factor ChIP-seq peak regions (TF ChIP-seq) comprise ChIP-seq binding profiles of 326 TFs in LoVo cells (Yan *et al*, 2013). The height of the signal indicates the number of different TFs binding at a particular region. TT-seq enhancer regions (black) and Med1-defined super-enhancers (gray (Yan *et al*, 2013)) are shown at the bottom.

B As in (A) but for *KLF5* (hg38; chr13:73,050,425–73,652,645).

© 2021 The Authors

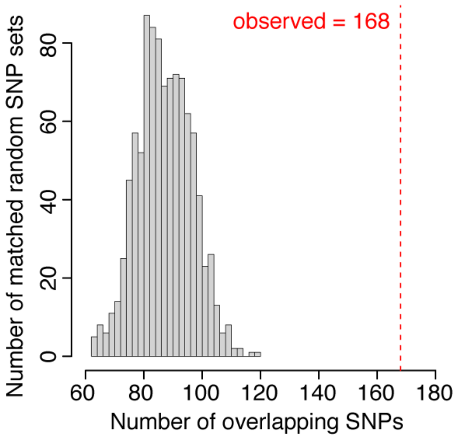

Figure EV5. Cancer-associated DNA sequence variation in transcribed enhancers.

Enrichment of non-coding cancer-associated single nucleotide polymorphisms (SNPs) in TT-seq-defined transcribed enhancer regions using randomly sampled matched non-coding SNPs from the 1000 Genomes project ((Auton *et al*, 2015) Materials and Methods). SNPs (n = 1,942) linked to the seven cancer types of this study were derived from the NHGRI-EBI genome-wide association studies (GWAS) catalog ((Welter *et al*, 2014) Materials and Methods). Histogram shows the number of overlaps observed in 1,000 matched random SNP sets. Dotted line shows the number of overlaps with non-coding cancer-associated GWAS SNPs.

© 2021 The Authors
