# Supplementary figures and images for: Transcriptionally active enhancers in human cancer cells
Source: Mol Syst Biol. 2021 Jan 27;17(1):e9873. doi: 10.15252/msb.20209873 (PMC7838827; doi:10.15252/msb.20209873)

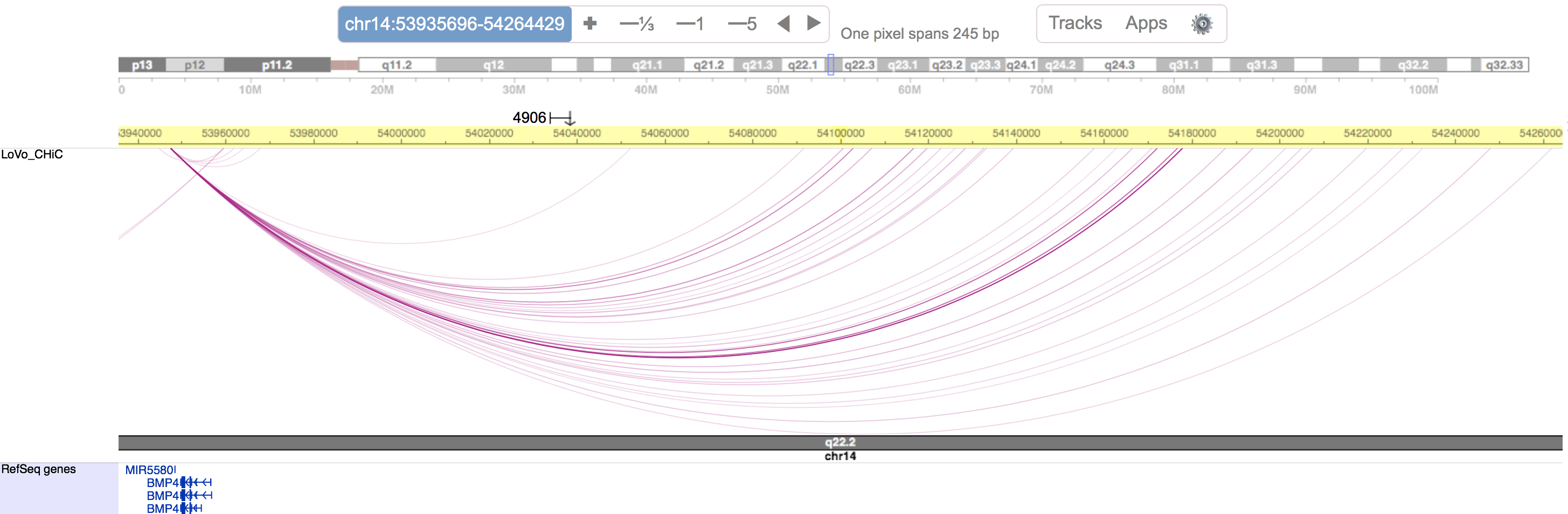

Supplement: Supplementary file 11 — Source Data for Figure 7 [file MSB-17-e9873-s010.zip › MSB-20-9873_SourceDataForFigure7/MSB-20-9873_SourceDataForFig7.png]

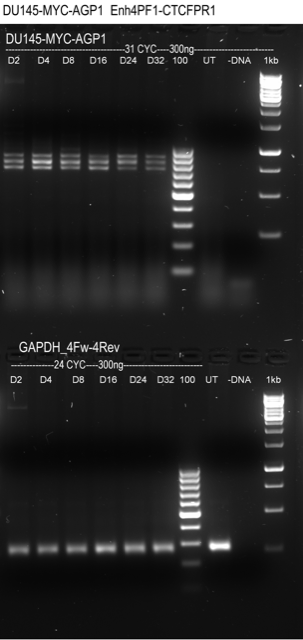

Supplement: Supplementary file 12 — Source Data for Expanded View [file MSB-17-e9873-s011.zip › MSB-20-9873_SourceDataForFigureEV2/MSB-20-9873_SourceDataForFigEV2_MYC.tiff]

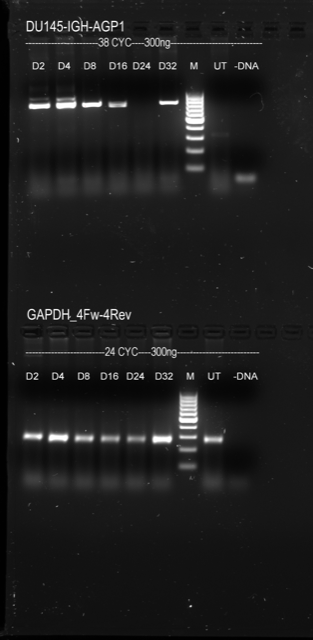

Supplement: Supplementary file 12 — Source Data for Expanded View [file MSB-17-e9873-s011.zip › MSB-20-9873_SourceDataForFigureEV2/MSB-20-9873_SourceDataForFigEV2_IGH.tiff]
